# Evolvable Acoustic Field Generated by a Transducer with 3D-Printed Fresnel Lens

**DOI:** 10.3390/mi12111315

**Published:** 2021-10-26

**Authors:** Danfeng Wang, Pengfei Lin, Zeyu Chen, Chunlong Fei, Zhihai Qiu, Qiang Chen, Xinhao Sun, Yan Wu, Lei Sun

**Affiliations:** 1 School of Mechanical and Electrical Engineering, Central South University, Changsha 410083, China; wangdf2019@126.com; 2 School of Microeletronics, Xidian University, Xi’an 710071, China; pflinxidian@163.com (P.L.); chenqiang1314021@163.com (Q.C.); xinhaosun@126.com (X.S.); w1210738083@163.com (Y.W.); 3 Interdisciplinary Division of Biomedical Engineering, The Hong Kong Ploytechnic University, Hong Kong 999077, China; zhihai.p.qiu@connect.polyu.hk (Z.Q.); lei.sun@polyu.edu.hk (L.S.)

**Keywords:** evolvable acoustic field, 3D printed Fresnel lens, finite element analysis, ultrasonic transducer

## Abstract

Evolvable acoustic fields are considered an effective method for solving technical problems related to fields such as biological imaging, particle manipulation, drug therapy and intervention. However, because of technical difficulties and the limited technology available for realizing flexible adjustments of sound fields, few studies have reported on this aspect in recent years. Herein, we propose a novel solution, using a Fresnel lens-focused ultrasonic transducer for generating excited-signal-dependent acoustic pressure patterns. Finite element analysis (FEA) is used to predict the performance of a transducer with a Fresnel lens. The Fresnel lens is printed using 3D additive manufacturing. Normalized intensity maps of the acoustic pressure fields are characterized from the Fresnel lens-focused transducer under various numbers of excited-signal cycles. The results demonstrate that under different cycle excitations, a temporal evolution acoustic intensity can be generated and regulated by an ultrasound transducer with a 3D Fresnel lens. This acoustical pattern control method is not only simple to realize but also has considerable application prospects.

## 1. Introduction

Ultrasonic field control technology, or ultrasonic focusing, is a key factor affecting the efficiency and performance of many widespread applications such as industrial nondestructive testing [1], ultrasonic ink jet printing technologies [2,3], medical ultrasonic therapy [4,5], drug manipulation [6,7] and ultrasonic imaging [8,9].

In recent years, many studies have discussed the method of diffraction-limited focusing by ultrasonic beam control technology using a multi-element array for phase modulation and correction [10,11,12]. Although this method can achieve effective sound field regulation, it is expensive and requires complex electronic equipment. Therefore, new methods for sound field regulation must be developed.

Currently, mechanical indentation is a commonly used focusing method that employs single-element focusing transducers [13,14] in applications, such as ball pressure exertion on a piezoelectric material [15,16,17,18]. However, this method has the drawback of possible element breakage during the shaping process. Another technique involves the acoustic lens [19,20,21], which consist of a refractive material with a curved interface analogous to an optical lens [22]. Recently, a single-element transducer with a 3D-printed holographic acoustic lens has proven capable of generating the designed static sound field [23]. However, the flexibility and machinability of the holographic acoustic lens are limited. Since the 1970s, Fresnel lenses have gained widespread application in the field of optics [24,25] and subsequently became a research hotspot [26,27,28,29] in acoustics. Earlier studies have demonstrated the focusing characteristics and efficiency of Fresnel lenses both in optical [30] and acoustic [31,32] applications. As in optics, the principle of the Fresnel sound lens is to design equidistant tooth patterns on one side of the lens and to achieve static focus of the specified range of sound waves through these tooth patterns.

In this paper, we report on the use of a Fresnel lens in a single-element focused ultrasonic transducer for generating sound pressure modes corresponding to specific excitation signals. The Fresnel lens was prepared by 3D printing and attached to the surface of a 5 MHz ultrasonic transducer manufactured through a conventional method. In our work, this new method was numerically analyzed using finite element modelling and characterized by a series of experiments. The simulation and experimental results both showed that this focusing method can achieve dynamic sound field regulation of single-element ultrasonic transducers; thus, it can be used to improve the quality of biological imaging and promote the development of applications such as drug release, particle manipulation and ultrasonic therapy.

## 2. Materials and Methods

### 2.1. Fabrication of the Fresnel Lens-Focused Ultrasonic Transducer

**Figure 1:**
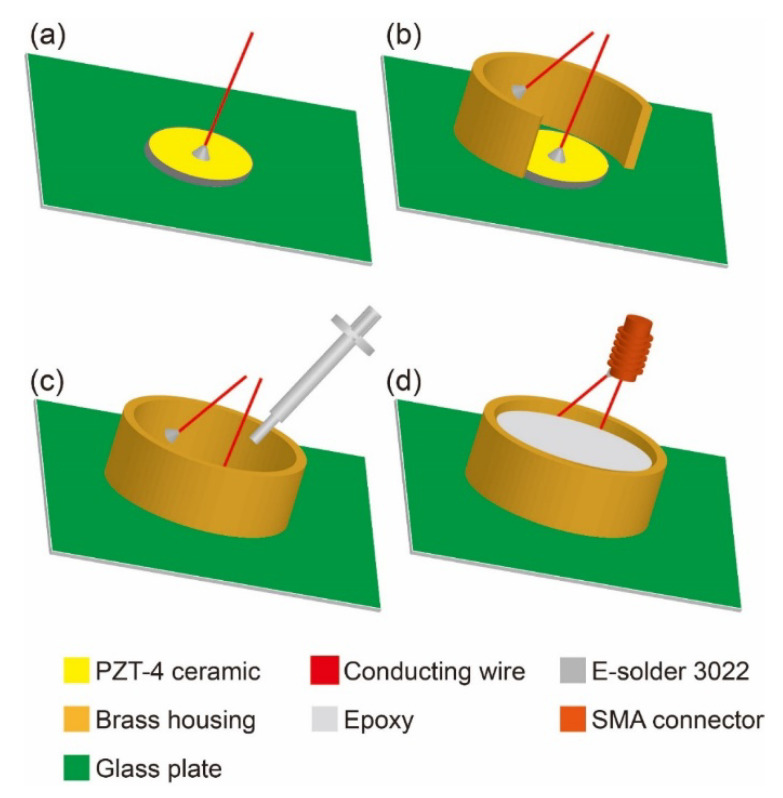
Schematic of the 5 MHz ultrasonic transducer manufacturing process (**a**–**d**).

Figure 1 shows the schematic of the 5 MHz ultrasonic transducer manufacturing process. In Figure 1a, the 5 MHz ultrasonic transducer, which was manufactured with conventional technology, using PZT-4 ceramic as piezoelectric material with a diameter of 25 mm, is shown with a conducting wire connected to the top surface of the PZT-4 ceramic with silver plating on both sides as the top electrode. Then, as shown in Figure 1b, a brass ring that was larger in diameter than the PZT-4 ceramic was placed surrounding the piezoelectric material as housing, and a conducting wire was connected to the inside of the brass ring to serve as the bottom electrode. Next, a quantity of epoxy (Tec 301) was injected into the brass ring with a syringe as a backing layer, as shown in Figure 1c. After curing for one day, the two wires were encapsulated into the SMA connector and connected to the external device, as shown in Figure 1d. Finally, gold was sputtered on the interface between the glass plate and the transducer within the brass housing using a gold sputter (Desk V HP TSC) to connect the bottom surface of the PZT-4 ceramic with the brass housing. A Fresnel lens (VeroClear Resin: velocity of sound, c = 2424 m/s, density, ρ = 1180 kg/m^3^, acoustic impedance, Z = 2.86 MRayl and attenuation, α = 5.5 dB/cm) was fabricated using a 3D-Stereolithography Apparatus printer (SLA, Form 2, Formlabs, Somerville, MA, USA) with a print-layer thickness of 25 μm. The lens profile was chosen to produce a focus at 38 mm from the surface using the geometric Fresnel lens design rules described by Mori et al. [33]. Acoustic Fresnel lenses exploit the fact that the phase is cyclical (modulo 2π); for instance, in the frequency domain, a phase of 3π is the same as that of π. The thickness of the lens at each point is set to introduce a specific phase delay to create a focal point at the target. Since the phase is kept between 0 and 2π radians using the modulo operation, the lens acquires the characteristic shape of a Fresnel lens. In this way, a lens gets compressed into a thinner format, which results in lower attenuation compared to a regular lens and enables closer focusing. The Fresnel lens obtained by this design is very thin, has lower attenuation than conventional lenses and easily achieves a close focus.

### 2.2. Experimental Setup and Characterization

During the experiments, we used an impedance analyzer system (Wayne Kerr Electronics, London, UK) to measure the frequency dependence of the magnitude and phase of the 5 MHz transducer. This transducer was connected to a JSR Ultrasonics pulser/receiver (DPR 500, Pittsford, NY, USA) to excite an electrical impulse at 200 Hz repetition rate and 50 Ω damping; pulse-echo response measurements were then conducted in distilled water. The energy involved was 12.4 μJ and no gain was applied. Subsequently, using an oscilloscope (RTE 1104, ROHDE&SCHWARZ, Munich, Germany), the frequency spectrum was computed by built-in fast FFT feature to display the echo response of the transducer. An X-cut quartz plate was used as a reflector.

According to Formulas (1) and (2), we can calculate the center frequency f_c_ and −6 dB bandwidth BW% of the 5 MHz PZT-4 ultrasonic transducer.

(1) $$f_{c}=\frac{f_{1}+f_{2}}{2}$$

(2) $$\mathrm{BW}\%=\frac{f_{2}-f_{1}}{f_{c}}\times 100\%$$

In the formula above, ${\;f}_{1}$ and $f_{2}$ are the frequency numerical values when the magnitude of the FFT of the echo drops to half of the peak value, and $f_{1}$ < $f_{2}$.

Finally, a 3D-ultrasound intensity measurement system (UMS3, Precision acoustics, Dorchester, UK) was used to measure the acoustic pressure field of the Fresnel lens-focused transducer. The Fresnel lens-focused transducer and a needle hydrophone (SN2010, 0.5 mm probe, Precision Acoustics, Dorchester, UK) were placed opposite each other in degassed water. The transducer was driven at the center frequency using a function generator combined with an RF power amplifier (A075, E&I, Rochester, NY, USA). The voltage applied to the transducer was 30 V. Signals from the hydrophone were captured by a digital oscilloscope (DSOX3024A, Agilent, Santa Clara, CA, USA) and then plotted in pseudo-color using MATLAB code.

### 2.3. Numerical Simulations

Numerical simulations of acoustic wave propagation in Fresnel lens structures and in water media were conducted using finite-element analysis software (PZFlex, Weidlinger Associates, Los Altos, CA, USA).

Because the transducer with the Fresnel lens is axisymmetric, we used a two-dimensional axisymmetric model in the finite element simulation, which allowed us to speed up the simulation and permitted us to select a higher simulation accuracy in this process. In the simulation system, the thickness of the PZT-4 ceramic was set to 400 µm to acquire the center frequency of 5 MHz. To fully absorb backward energy, the backing layer material selected was 1 mm epoxy resin. During the simulation process, the piezoelectric material was connected in series to a 50 Ω resistor, and the transducer was excited with different cycles of sinusoidal signal: an excitation frequency of 5 MHz, and a driving voltage of 1 V peak-to-peak. The mesh size was chosen to be 1/15 of the wavelength in all directions. The parameters of the piezoelectric materials (PZT-4) and other materials used in the PZFlex simulation are listed in Table 1 and Table 2, respectively. Because of the symmetry of PZT-4, the matrix of elastic constant (c^E^), relative permittivity (ε) and piezoelectric coefficient (e) of PZT-4 ceramic are as follows:

$$c^{E}=\left(\begin{array}{cccccc} c_{11}^{E} & c_{12}^{E} & c_{13}^{E} & 0 & 0 & 0\\ c_{12}^{E} & c_{11}^{E} & c_{13}^{E} & 0 & 0 & 0\\ c_{13}^{E} & c_{13}^{E} & c_{33}^{E} & 0 & 0 & 0\\ 0 & 0 & 0 & c_{44}^{E} & 0 & 0\\ 0 & 0 & 0 & 0 & c_{44}^{E} & 0\\ 0 & 0 & 0 & 0 & 0 & c_{66}^{E} \end{array}\right)$$

$$\varepsilon =\left(\begin{array}{c} \varepsilon_{11}\\ 0\\ 0 \end{array}\begin{array}{c} 0\\ \varepsilon_{11}\\ 0 \end{array}\begin{array}{c} 0\\ 0\\ \varepsilon_{33} \end{array}\right)$$

$$e=\left(\begin{array}{c} 0\\ 0\\ e_{31} \end{array}\begin{array}{c} 0\\ 0\\ e_{31} \end{array}\begin{array}{c} 0\\ 0\\ e_{33} \end{array}\begin{array}{c} 0\\ e_{15}\\ 0 \end{array}\begin{array}{c} e_{15}\\ 0\\ 0 \end{array}\begin{array}{c} 0\\ 0\\ 0 \end{array}\right)$$

## 3. Results

### 3.1. Numerical Studies of Ultrasonic Transducer with Fresnel Lens

**Figure 2:**
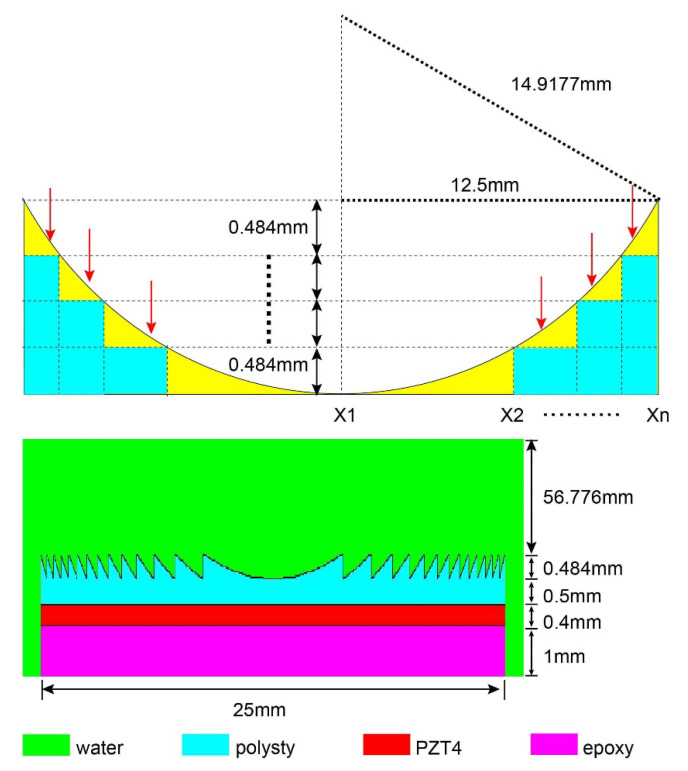
Designed specification of the Fresnel lens-focused ultrasonic transducer.

Figure 2 shows the design specification for the Fresnel lens-focused ultrasonic transducer. We started by employing a 5 MHz ultrasonic transducer with a diameter of 25 mm and a lens with f_#_ = 0.6 (f_#_ is the ratio of the radius of curvature of the lens to the diameter of the ultrasonic transducer) as the starting point to design the novel lens. The f_#_ was calculated according to Formula (3).

(3) $$f_{\#}=\frac{R}{d}$$

where R and d are radius of curvature of lens and diameter of piezoelectric element.

At a design frequency of 5 MHz, the wavelength in the lens is 0.484 mm. We divided the region along the wave propagation direction by an integer multiple of the wavelength, and then subtract the maximum integer wavelength thickness in each region to form the novel lens.

**Figure 3:**
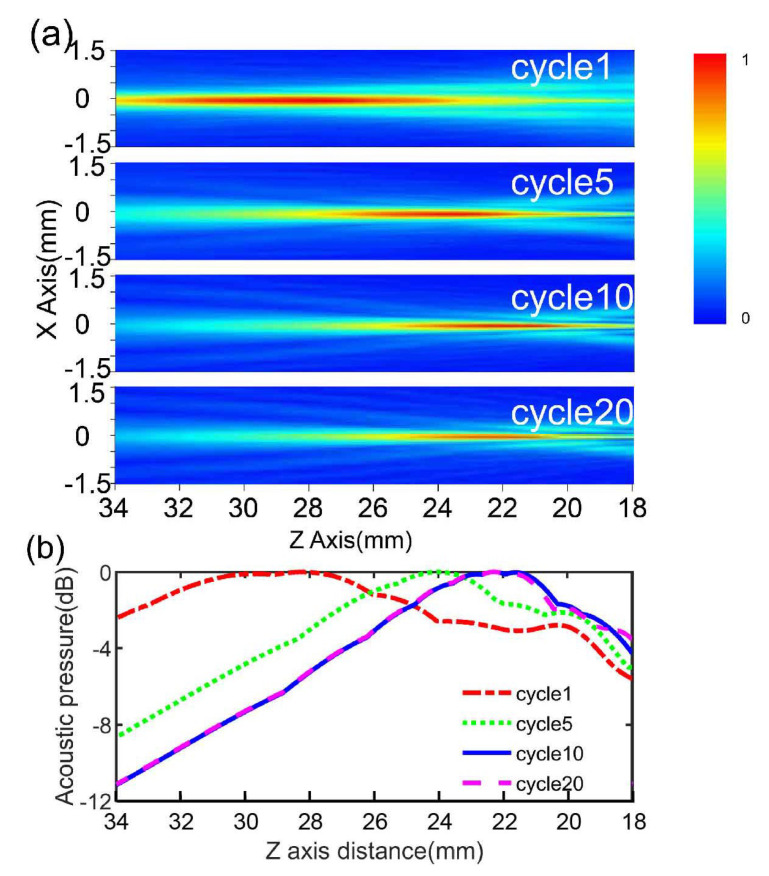
(**a**) Focal depth determined from acoustic pressure patterns and (**b**) acoustic pressure along the Z axis under different cycle numbers, by PZFlex simulation.

In the simulation process, the acoustic pressure patterns determined the focal depth, as shown in Figure 3a. The color scale indicates normalized sound pressure, which gradually increases from blue to red. As depicted, the acoustic pressure patterns of the device change with the number of excitation signal cycles. As the number of excitation signal cycles increases, the focus region (red region) gradually shifts towards the lens surface until the cycle number is greater than 10, after which this number remains unchanged as the number of excitation cycles increases. Figure 3b shows the acoustic pressure along the Z axis under different cycle numbers obtained from the PZFlex simulation and processed through MATLAB. Assuming a value of 1490 m/s for the speed of sound and that the focus points with maximum pressure are those with a value of 0 dB, the focal depth of the focus points can be obtained from the PZFlex simulation. The results show that the focal depth changes from 28.2 to 22.28 mm when the cycle numbers increase from 1 to 10, after which it remains unchanged as the cycle number increases, as shown in Figure 3b. The −6 dB beam widths of the device with 1, 5, 10 and 20 cycles were determined as 0.86, 0.62, 0.5 and 0.5 mm, respectively. Based on the results of FEM, we can conclude that the Fresnel lens-focused transducer has a tight focus, and its focus characters change with the excitation signal.

### 3.2. Experiments on Fresnel Lens-Focused Ultrasonic Transducer

**Figure 4:**
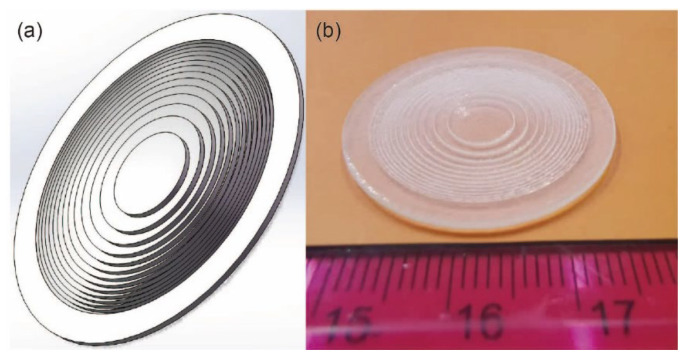
(**a**) Structure diagram and (**b**) Physical figure of Fresnel lens.

The Fresnel lens structure drawing produced by SolidWorks software is shown in Figure 4a. And the actual physical figure of this Fresnel lens, 3D-printed also using SolidWorks software is shown in Figure 4b. The effective area diameter of the Fresnel lens was 25 mm.

**Figure 5:**
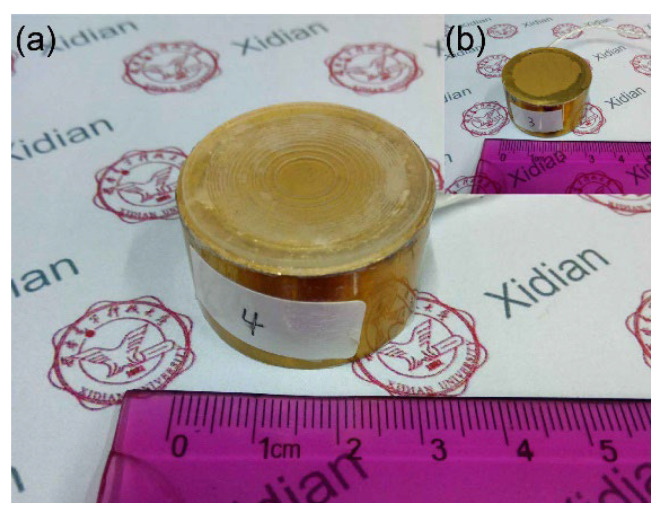
(**a**) Transducer with Fresnel lens and (**b**) without Fresnel lens.

Figure 5a,b show the transducer with and without a Fresnel lens, respectively. The transducers were fabricated with traditional production methods and the Fresnel lens was bonded to the transducer using 301 Epoxy (Epoxy Technology, Billerica, MA, USA).

**Figure 6:**
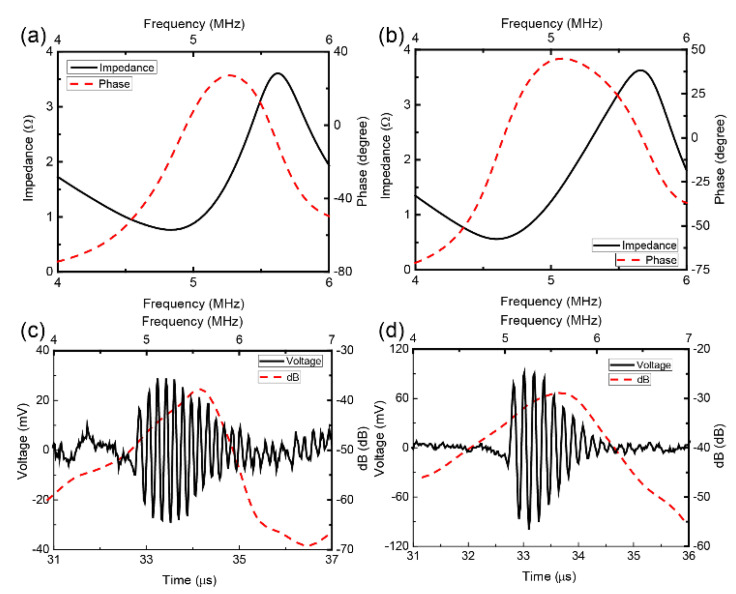
Top, frequency dependence of electrical impedance and phase plots of (**a**) the transducers with Fresnel lens and (**b**) without Fresnel lens; Bottom, measured pulse-echo waveform with normalized frequency spectrum of (**c**) the transducers with Fresnel lens and (**d**) without Fresnel lens.

Figure 6a,b displays the frequency dependence of electrical impedance and phase plots of the transducers with and without Fresnel lens. The resonant frequency and its corresponding impedance were determined from the plots as 5.28 MHz and 1.8 Ω with the lens, and 5.1 MHz and 1.58 Ω without the lens. The measured pulse-echo waveform with normalized frequency spectrum is shown in Figure 6c,d. Here, it was determined that the center frequency is 5.46 MHz with lens and 5.48 MHz without lens, and the −6 dB bandwidth was measured to be 11.54% with lens and 18.98% without lens. The decline of −6 dB bandwidth of the transducers with Fresnel lens is attributed to the bonded lenses, which degraded reception performance. Given that this study is focused on the performance of Fresnel lens transducers with regards to sound field control, this reduction of −6 dB bandwidth is considered to have little effect on this.

**Figure 7:**
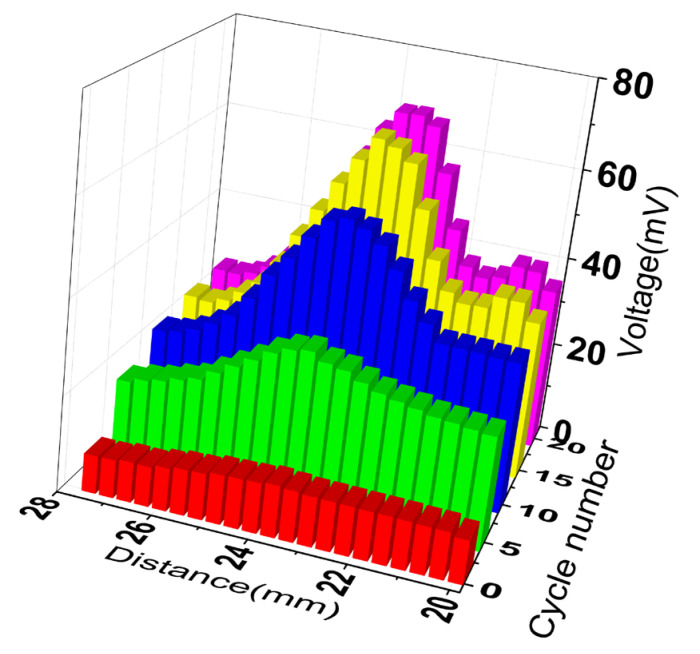
The peak-to-peak voltages at different locations under different excitation numbers.

With the purpose of studying its sound field regulation effect, the peak-to-peak voltages at different locations under different excitation numbers were measured, as shown in Figure 7. The envelope of the histogram under different cycle numbers is different, which shows that the sound field distribution changes.

**Figure 8:**
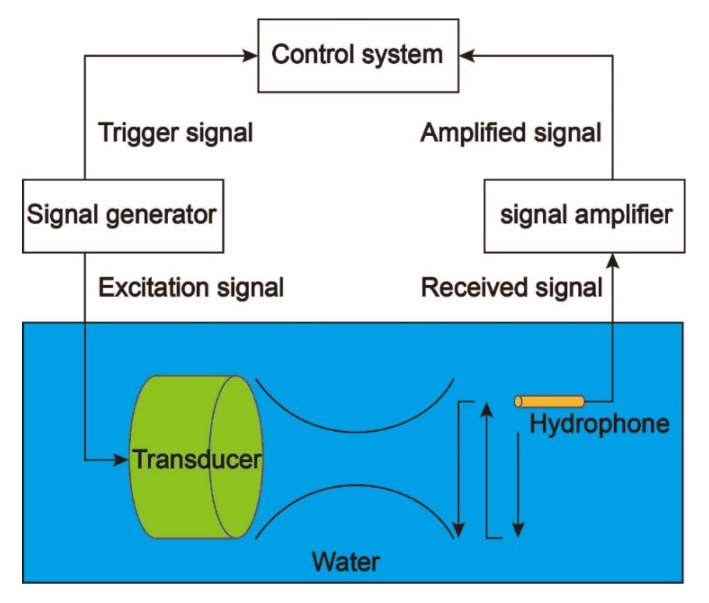
Schematic diagram of 3D ultrasound intensity measurement system.

It is worth mentioning that to eliminate the possibility of human error in these measurements, all acoustic pressure maps were acquired using 3D ultrasound intensity measurement devices. A schematic diagram of the 3D ultrasound intensity measurement system used is depicted in Figure 8. In such a system, we control the signal generator first, to transmit the required excitation signal to the transducer with a Fresnel lens. At the same time, the signal generator emits a trigger signal that is used by the control system to modulate the hydrophone for the corresponding acoustic pressure signal. Finally, the control system processed the data and generated a sound field distribution map.

**Figure 9:**
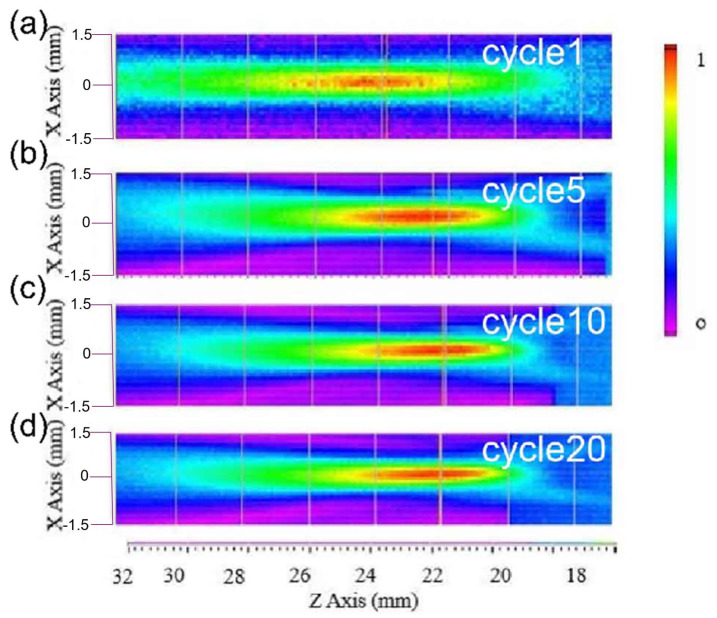
(**a**–**d**) Normalized intensity maps of the pressure fields from the Fresnel lens-focused transducer with different cycle numbers of excited signal.

The normalized intensity maps of the pressure fields from the Fresnel lens-focused transducer with different cycle numbers of excited signals are shown in Figure 9a–d. The color scale again indicates normalized sound pressure, which gradually increases from purple to red. The focusing effect of the Fresnel lens is very evident, as indicated by the focal spots in Figure 9a–d.

Furthermore, referring to the intensity maps in the x-z plane in Figure 9a–d, it can be seen that the focal spot shifts closer to the lens surface until the cycle number is greater than 10, after which it remains unchanged, as was expected from the simulations. This tendency of the focal length to change with different numbers of excitation pulses confirms the same effect described in the simulation shown in Figure 3. The differences between the simulation and experiment may be attributed to the number of cycles used. Another explanation for such differences is that we received and displayed the echo from the Fresnel lens-focused transducer—the excited signal has only one cycle, while the received signal shows several cycles owing to the performance of transducer.

**Figure 10:**
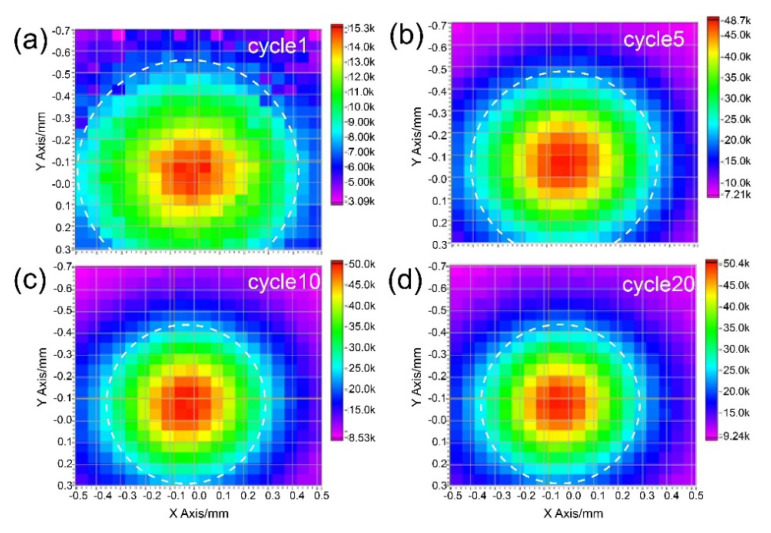
(**a**–**d**) Intensity maps of the pressure fields on the focus plane from the Fresnel lens-focused transducer with different cycle numbers of excited signal.

The intensity maps of the pressure fields on the focus plane from the Fresnel lens-focused transducer with different cycle numbers of excited signal are shown in Figure 10a–d. The color scale here indicates the sound pressure intensity, which gradually increases from purple to red. The resolution of the image is approximately 50 μm. In the focus plane, we used the white dotted circle to draw the area where the sound pressure is half of the maximum sound pressure. Then, the −6 dB beam widths of the device with different cycles can be expressed by the diameter of the circle. As shown in Figure 7, the diameter of the circle decreases as the cycle number of excited signal increases from 1 to 10, and the diameters of the circle are almost unchanged as the cycle number of excited signal increases from 10 to 20, which is consistent with the simulation results.

## 4. Discussion and Conclusions

Limited by commonly used technology, under a specific transducer design, the acoustic field is fixed. Ahmet et al. demonstrated that using a Fresnel lens, it is possible to focus 25 kHz acoustic waves to manipulate small particles. However, it is also shown that there are standing waves between the transducer and its acoustic lens, which can reduce the output energy [6]. To obtain a tunable focus acoustic lens, a nonlinear acoustic lens has been designed and shown to be useful in the low frequency range of 0.1–180 kHz, but it only worked for high-energy applications [34].

In this study, the use of a Fresnel lens-focused ultrasonic transducer is reported for generating sound pressure patterns associated with specific excitation signals, by combining a 5 MHz central frequency ultrasonic transducer with a lens that can eliminate standing waves. The normalized intensity diagram of the acoustic pressure field from the Fresnel lens-focused transducer under various cycle numbers of excited signal was measured. The experimental results demonstrated the focal depth changed from 23.8 to 21.9 mm when the number of excited signal cycles increased from 1 to 20, and remained unchanged after 10 cycles, which is consistent with the simulation results. The reason behind the focal depth remaining unchanged as the cycle number increases beyond 10 is that, as the number of pulses increases to 10 while a new period of pulses generates vibrations, the vibrations of earlier period pulses can be neglected, and the number and intensity of vibrations that make up the maximum sound pressure remain unchanged. The experimental results also showed that the −6 dB beam widths of the transducer with the Fresnel lens decreased when the cycle numbers of excited signal increased from 1 to 20, and remained unchanged after 10 cycles, which is also consistent with the simulation results. These results demonstrate that under different cycle excitation, a temporal evolution acoustic intensity at various longitudinal locations along the focus can be generated and controlled by a 3D printed Fresnel lens-focused ultrasound transducer. This simple ultrasonic field control (focusing) technology has broad application prospects in biomedical and other fields.

## Figures and Tables

**Table 1:** Piezoelectric materials used for the PZFlex simulation.

Parameter	Value	Parameter	Value
$\rho \;\left(\mathrm{kg}/m^{3}\right)$	7500	$c_{11}^{E}\;\left(10^{10}\;N/m^{2}\right)$	14.7
$e_{31}\;\left(C/m^{2}\right)$	−3	$c_{12}^{E}\;\left(10^{10}\;N/m^{2}\right)$	8.11
$e_{33}\;\left(C/m^{2}\right)$	16.7	$c_{13}^{E}\;\left(10^{10}\;N/m^{2}\right)$	8.11
$e_{15}\;\left(C/m^{2}\right)$	11.4	$c_{33}^{E}\;\left(10^{10}\;N/m^{2}\right)$	13.2
$\varepsilon_{11}$	730	$c_{44}^{E}\;\left(10^{10}\;N/m^{2}\right)$	3.13
$\varepsilon_{33}$	635	$c_{66}^{E}\;\left(10^{10}\;N/m^{2}\right)$	3.06

**Table 2:** Other materials used in the PZFlex simulation.

Material	Function	c (m/s)	ρ (kg/m^3^)	Z (MRayls)
VeroClear Resin	Lens	2424	1180	2.86
Water	Front load	1490	1000	1.49
Epoxy	Backing layer	2080	2849	5.92
